# Supplementary material for: Chitin Deacetylase, a Novel Target for the Design of Agricultural Fungicides
Source: J Fungi (Basel). 2021 Nov 25;7(12):1009. doi: 10.3390/jof7121009 (PMC8706340; doi:10.3390/jof7121009)
Supplement: Supplementary file 1 [file jof-07-01009-s001.zip › Figure legends.pdf]

Figure S1. *In planta* expression of HIGS constructs and target genes. Total RNA was isolated from agroinfiltrated melon cotyledons and the expression of HIGS constructs and target genes was analyzed by RT-qPCR. (A) Expression of HIGS constructs. The relative expression of HIGS constructs PxCDA-HIGS and CmCERK1-HIGS was analyzed 24 h after agro-infiltration in absence of the fungal pathogen. Relative transcription levels of HIGS constructs were normalized to the transcription of the endogenous control melon *CmACT7* gene (XM_008462689.2). (B) Expression of target genes upon HIGS. The relative expression of *P. xanthii* transcripts *PxCDA1* and *PxCDA2*, and the melon chitin receptor gene *CmCERK1*, was analyzed 24 h after pathogen inoculation. Relative transcription levels of fungal and plant transcripts were normalized to the transcription of the endogenous control genes *P. xanthii PxTUB2* (KC333362) and melon *CmACT7*, respectively. Bars indicate the means ± standard errors of three technical replicates from three different samples, each from five pooled cotyledons.

Figure S2. Representative images of leaf disc assays. Melon cotyledon discs were treated with the different compounds and inoculated with *P. xanthii* as described in Material and Methods. **(A)** Disease-suppressing effect of carboxylic acids against cucurbit powdery mildew. **(B)** Non-disease suppressing effect of other chelating agents.
