# Supplementary material for: Chitin Deacetylase, a Novel Target for the Design of Agricultural Fungicides
Source: J Fungi (Basel). 2021 Nov 25;7(12):1009. doi: 10.3390/jof7121009 (PMC8706340; doi:10.3390/jof7121009)
Supplement: Supplementary file 1 [file jof-07-01009-s001.zip › Table S1.pdf]

Table S1. Primers used in this study.

Primer name	Sequence (5´- 3´)[1]
Plasmid construction	
PxCDAsi-F	AAAAAGCAGGCTCTCGGCCCAAGTATTGGAAGCTGTGCTG
PxCDAsi-R	AGAAAGCTGGGTGCTGTTACTGCCCTGTAGCCCGAAGAC
CERK1si-F	AAAAAGCAGGCTCTGGGAGGGTGCCAATTTGTCTTTCG
CERK1si-R	AGAAAGCTGGGTGTGGCACCTACTACAAGGAGAAACC
*att*B1	GGGGACAAGTTTGTACAAAAAAGCAGGCT
*att*B2	GGGGACCACTTTGTACAAGAAAGCTGGGT
Expression analysis of HIGS constructs and target genes	
CDA-HIGS-F	CGGCCCAAGTATTGGAAGCTGTGCTG
CDA-HIGS-R	CTGTTACTGCCCTGTAGCCCGAAGAC
CERK1-HIGS-F	GGGAGGGTGCCAATTTGTCTTTCG
CERK1-HIGS-R	GGCACCTACTACAAGGAGAAACC
ACT7-F	TCATGGTGGGTATGGGCCAGAAAG
ACT7-R	TACAGTGATAGAACGGCCTGGATG
CDA1-F	ATGCAGATGTTAAATTTGGCACGTACGG
CDA1-R	TGTATAAGAGATGACCACTGAC
CDA2-F	ATGCAGATGTTAAATTTGGCACGTACGG
CDA2-R	TGTCCACTGGTATTTGTAGTGTC
TUB2-F	GATTTAGAGCCCGGAACAATGGACG
TUB2-R	CACATCCTTCAGCTTCACGCCGAAC
CERK1-F	TGGTCAAGGTGATGGTGGTATAGC
CERK1-R	TCGACCTGTGCCTCGTAAATGTTG

[1]Underlined sequence is the adapter for *att*B1 or *att*B2 primers
