# Supplementary figures and images for: Chitin Deacetylase, a Novel Target for the Design of Agricultural Fungicides
Source: J Fungi (Basel). 2021 Nov 25;7(12):1009. doi: 10.3390/jof7121009 (PMC8706340; doi:10.3390/jof7121009)

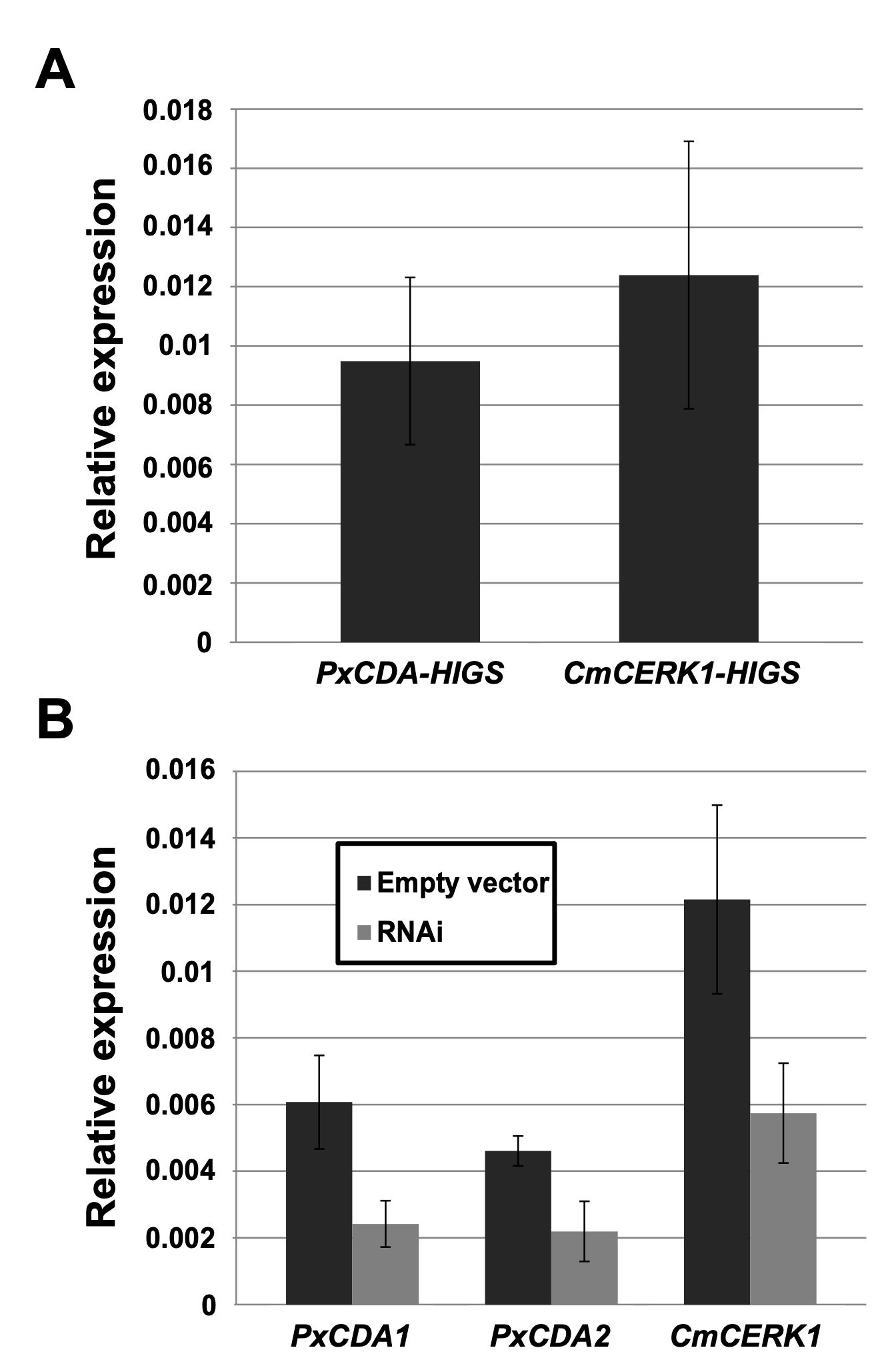

Supplement: Supplementary file 1 [file jof-07-01009-s001.zip › Figure S1.png]

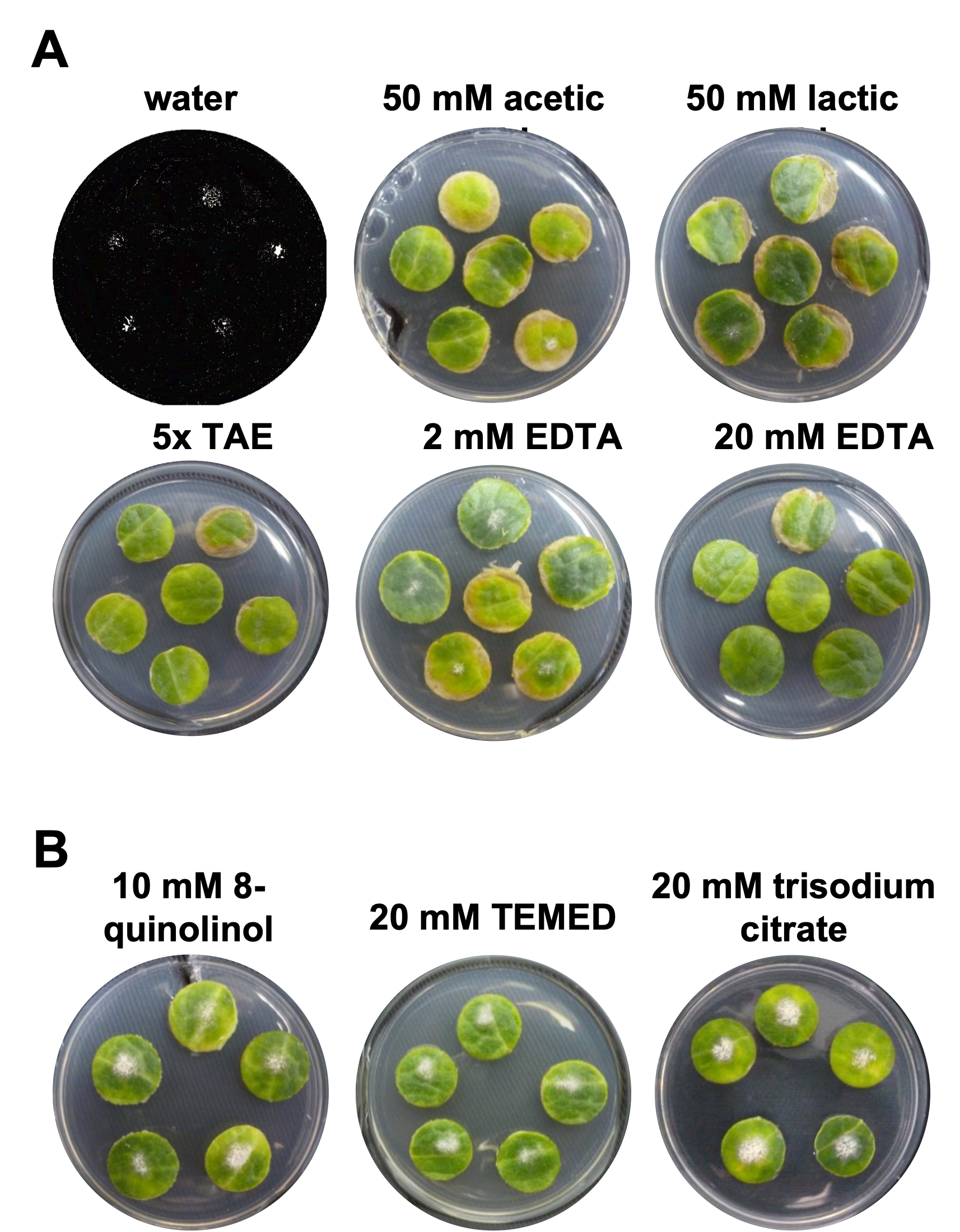

Supplement: Supplementary file 1 [file jof-07-01009-s001.zip › Figure S2.png]
